# Evolution of Tropical Cyclone Properties Across the Development Cycle of the GISS‐E3 Global Climate Model

**DOI:** 10.1029/2021MS002601

**Published:** 2022-01-05

**Authors:** Rick D. Russotto, Jeffrey D. O. Strong, Suzana J. Camargo, Adam Sobel, Gregory S. Elsaesser, Maxwell Kelley, Anthony Del Genio, Yumin Moon, Daehyun Kim

**Affiliations:** ^1^ Lamont‐Doherty Earth Observatory Columbia University Palisades NY USA; ^2^ Now at Gro Intelligence New York NY USA; ^3^ Now at AIR Worldwide Boston MA USA; ^4^ Department of Applied Physics and Applied Mathematics Columbia University New York NY USA; ^5^ NASA Goddard Institute for Space Studies New York NY USA; ^6^ SciSpace LLC New York NY USA; ^7^ Department of Atmospheric Sciences University of Washington Seattle WA USA

**Keywords:** tropical cyclones, tropical meteorology, hurricanes, climate model

## Abstract

The next‐generation global climate model from the NASA Goddard Institute for Space Studies, GISS‐E3, contains many improvements to resolution and physics that allow for improved representation of tropical cyclones (TCs) in the model. This study examines the properties of TCs in two different versions of E3 at different points in its development cycle, run for 20 years at 0.5° resolution, and compares these TCs with observations, the previous generation GISS model, E2, and other climate models. E3 shares many TC biases common to global climate models, such as having too few tropical cyclones, but is much improved from E2. E3 produces strong enough TCs that observation‐based wind speed thresholds can now be used to detect and track them, and some storms now reach hurricane intensity; neither of these was true of E2. Model development between the first and second versions of E3 further increased the number and intensity of TCs and reduced TC count biases globally and in most regions. One‐year sensitivity tests to changes in various microphysical and dynamical tuning parameters are also examined. Increasing the entrainment rate for the more strongly entraining plume in the convection scheme increases the number of TCs (though also affecting other climate variables, and in some cases increasing biases). Variations in divergence damping did not have a strong effect on simulated TC properties, contrary to expectations based on previous studies. Overall, the improvements in E3 make it more credible for studies of TC activity and its relationship to climate.

## Key Points

- The new NASA GISS‐E3 global climate model at 0.5° resolution simulates more realistic tropical cyclone activity versus the E2 version
- Hurricane intensity storms are now simulated, and the average storms are comparable to other 0.5° climate models
- Tropical cyclone counts in E3 are very sensitive to convective plume entrainment rates but not to divergence damping coefficients

## 1 Introduction

Tropical cyclones (TCs) are among Earth's most potent natural hazards affecting life and property. There is much interest in how these storms will change as a consequence of anthropogenic global warming, but predicting these changes with global climate models (GCMs) has proven challenging due in part to the dependence of TC dynamics on processes that are unresolved at such models' typical spatial resolutions. All else being equal, higher horizontal resolution in GCMs typically improves TC activity characteristics (Moon, Kim, Camargo, Wing, Sobel, et al., 2020; Murakami & Sugi, 2010; Shaevitz et al., 2014; Vidale et al., 2021; Walsh et al., 2013; Wehner et al., 2015), but models with similar resolutions can still vary widely in their representations of TC activity (Camargo et al., 2020), as differences in the model configuration such as the convection scheme (Duvel et al., 2017; Kim et al., 2012; Murakami et al., 2012; Vitart et al., 2001), dynamical core (Reed et al., 2015), and coupling to the ocean (Li & Sriver, 2018; Scoccimarro et al., 2017; Zarzycki, 2016) also affect TC properties. Given these differences between GCMs, and the rapid increase of computational expense with resolution, some progress in understanding changing TCs in future climates is being gained using models run at resolutions of about half a degree (currently, a plausible compromise between computational expense and TC simulation fidelity), in single‐model large ensembles (Yoshida et al., 2017) and in multi‐model ensembles including the U.S. Climate Variability and Predictability Program (CLIVAR) Hurricane Working Group experiments (Shaevitz et al., 2014; Walsh et al., 2015) and HighResMIP (Haarsma et al., 2016; Roberts et al., 2020a, 2020b), an endorsed project of the Coupled Model Intercomparison Project (CMIP) Phase 6 (Eyring et al., 2016).

To better understand the behavior of TCs in such multi‐model ensembles predicting future climates, it is helpful to analyze TCs in depth in the individual GCMs. The questions of how TCs are affected as models go through their development cycles, and the sensitivities of TC activity and other climate variables to the parameters often used to tune GCMs, are also of interest.

Here we characterize the properties of TCs in two development versions of the NASA Goddard Institute for Space Studies (GISS) ModelE3, the next generation GCM still under development at GISS: one from early in the model's development and previously used by Cesana et al. (2019) and Camargo et al. (2020), and another after a further year of development. The previous generation GISS GCM, ModelE2 (Schmidt et al., 2014), has no version with horizontal resolution finer than 1°, and lags behind other GCMs, even those of similar resolutions, in the intensity of its simulated TCs (Camargo et al., 2016; Shaevitz et al., 2014). A major goal of this study is to evaluate whether the simulation of TCs in the 0.5° version of E3 is now comparable to that of other GCMs (e.g., Camargo et al., 2020; Roberts et al., 2020a; Shaevitz et al., 2014) and the extent to which this assessment has changed through the model development process. E3 is one of several components of GISS's contributions to CMIP6, along with E2.1 (Kelley et al., 2020) and the high‐top version E2.2 (Orbe et al., 2020; Rind et al., 2020), all successors of E2, which was GISS's contribution to CMIP Phase 5 (Taylor et al., 2012). E3 differs from these other versions in that it uses a cubed sphere grid with the Putman and Lin (2007) dynamical core. In contrast to the E3 version being used for CMIP6, which uses a C90 grid, here we consider the C180 version of the cubed sphere, in which each face of the cube measures 180 grid cells in length; this is equivalent to about 0.5° or 55 km resolution. This is the first in depth analysis of TC activity in the high‐resolution version of E3. By comparison, Camargo et al. (2016) analyzed TCs in a version of E2 with a C90 cubed sphere grid and the dynamical core of Suarez and Takacs (1995).

In addition to making comparisons between the two versions of E3 analyzed here, and between these and observations, we are also interested in understanding how the GISS TCs depend on the model physics and the extent to which various parameters can be tuned to improve TC representation. To this end, we have run numerous short experiments with E3 in which individual tuning parameters were changed, and we examine the resulting changes in TC properties as well as other climate variables and the errors in these variables with respect to observations. These are inspired by past experiments showing the sensitivities of TC properties to entrainment rate parameters in E2 (Kim et al., 2012) and the scale‐selective damping rate of divergent horizontal flow in the Geophysical Fluid Dynamics Laboratory (GFDL) High‐Resolution Atmospheric Model (HiRAM) (Zhao et al., 2012). These help us understand how much room there might be to further improve TCs in E3 through tuning at the same resolution, and whether such improvements would come at the cost of making other aspects of the climate less realistic. Our preference for the sensitivity experiments was to cover a larger span of parameters and their values in one‐year simulations, instead of fewer experiments with longer simulations. While longer simulations would be preferable, the model biases are large enough that they are easily apparent in one‐year simulations, and the sensitivity experiments clearly identify which of the parameters tested have the strongest influences on the simulated TCs.

In Section 2 we describe the model versions, observational data sources, and the methods of our data analysis. Section 3 contains statistics of the overall distribution of storms, genesis, and Accumulated Cyclone Energy, globally, regionally and across in the seasonal cycle. Section 4 explores the physical properties of the simulated tropical cyclones, such as maximum wind speed, minimum central pressure, and lifetime, and the storms' spatial structure. Section 5 discusses the TC and climate responses to the 1‐year tuning experiments. Section 6 summarizes our work and discusses possibilities for further improvement of TC representation.

## 2 Methodology

Version 1 (V1) of the model is an early version of E3 that was used for NASA's contribution to the study by Camargo et al. (2020) on whether there are cross‐model relationships between TC climatology and environmental variables and for the study of tropical low cloud responses to sea surface temperature (SST) forcings by Cesana et al. (2019). Version 2 (V2) is a refined version of the model after a further year of model development. Structurally, V2 differs from V1 primarily in its treatment of stratiform cloud microphysics, adding growth of snow by vapor deposition omitted from the scheme of Gettelman and Morrison (2015) and correcting several errors in the scheme as originally implemented in V1. Altogether, these updates influence tropical upper‐tropospheric relative humidity and latent heating, but we have not attempted to systematically quantify each effect individually. Also, in V1, inconsistent graupel densities were used in different parts of the convective ice parameterization, and this discrepancy has been corrected in V2. Convective precipitation drop size distributions were also modified, which resulted in larger precipitation effective radii. These changes in convective condensate characteristics led to a roughly 5%–10% increase in cloud liquid water path, and a roughly 1–5 W m^−2^ increase in top of atmosphere (TOA) reflected shortwave (SW) radiation, varying regionally. The broad increase in reflected SW radiation contributed to a larger TOA energy imbalance found in V2, which is discussed in Section 5.

The two model versions were run with default entrainment and divergence damping parameters from 1980 to 2000, forming 21 Northern Hemisphere TC seasons (January to December) and 20 complete seasons for the Southern Hemisphere (July to June) and are referred to as 20‐year test runs. Most of this paper analyzes these test runs, while Section 5 analyzes TC and climate properties in 1‐year sensitivity experiments in which various entrainment and damping parameters were modified. The models were run in their atmosphere‐only configurations, with specified, monthly varying SSTs from 1980 to 2000 taken from the Met Office Hadley Centre SST data set version 2.1 (Titchner & Rayner, 2014). The 1‐year sensitivity experiments were run for the year 1990, chosen for being an El Niño–Southern Oscillation neutral year.

For our database of observed TCs, we use the International Best Track Archive for Climate Stewardship (IBTrACS; Knapp et al., 2010; Kruk et al., 2010), using in particular the data reported by the U.S. agencies: the National Hurricane Center for the North Atlantic and Eastern North Pacific and the Joint Typhoon Warning Center for the Western North Pacific, South Pacific, and Indian Oceans. For the observations, we only include best track data at 0, 6, 12, or 18 hr UTC, and we filter the storms so that only tropical cyclones that reach at least tropical storm strength (34‐knot sustained winds, 1 min average) are included. However, portions of the lifetimes of these TCs with the wind speed below 34 knots are included in the statistics. We include all portions of these tracks due to the inconsistency in labeling storms as extratropical or post tropical across basins, even within the U.S. agencies.

TCs in the model simulations are tracked using the method described in Zhao et al. (2009), with specific options chosen as follows. A 12‐model‐grid‐box‐wide window (the standard in the code for C180 grids) is looped through each 6‐hourly model output snapshot to detect possible tropical cyclones. Each TC candidate is required to exceed an 850 hPa cyclonic relative vorticity threshold of 3.5 × 10^−5^ s^−1^, to have a positive warm core temperature anomaly relative to the search window mean (averaged between 300 and 500 hPa at the temperature maximum), have a sea level pressure minimum within 4° of the vorticity maximum, and to be located between 70°N and 70°S. Once identified, the storm candidates are tracked forward in time searching within 1,500 km of the previous position (defined as the location of the SLP minimum), and further criteria are applied for tracks to be included. TCs must, on three separate (not necessarily consecutive) days, exceed the cyclonic 3.5 × 10^−5^ s^−1^ vorticity threshold, have a warm core temperature anomaly exceeding 1°C, and have maximum surface wind speed, defined here as the wind speed at 10 meters in the postprocessed model output, exceeding 15.2 m/s (but the winds can drop below this threshold at times during the trajectory). In addition, the 10 m winds must at some point exceed 17.0 m/s, or tropical storm intensity. As pointed out by, for example, Sugi et al. (2017) and Yoshida et al. (2017), since the maximum wind speed decreases with coarser model resolution, it is common to use a lower threshold than the World Meteorological Organization definition of 17.0 m/s when tracking TCs in GCMs. Similarly to other studies at this resolution (e.g., Zhao et al., 2009), we do not impose a threshold lower than 17.0 m/s for the lifetime maximum wind speed in order to obtain a reasonable number of TCs to analyze, but we relax this slightly for the longevity determination to avoid excluding storms that only briefly exceed 17.0 m/s. The choice of the lifetime maximum wind speed threshold strongly affects the number of weaker storms detected, but does not affect stronger storms. The choice of tracking scheme can also affect the number of TCs detected in GCM output (Aarons et al., 2021; Horn et al., 2014; Roberts et al., 2020a), even after differences in detection thresholds are made uniform across schemes (Horn et al., 2014). Our focus is less on the total number of TCs than on their physical characteristics and the ability to simulate storms of hurricane strength.

**Figure 1:**
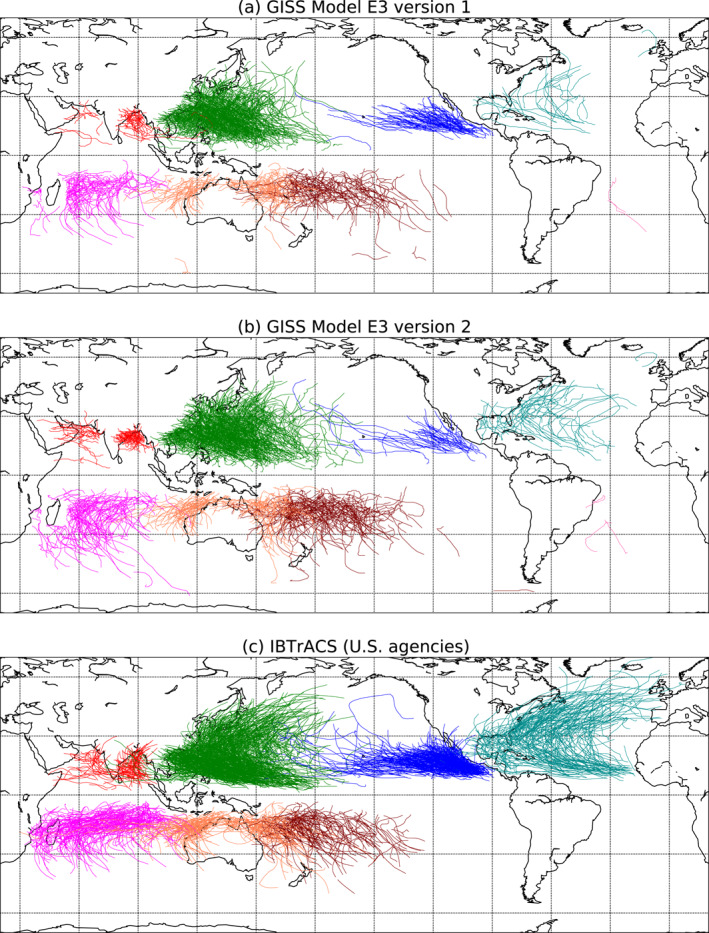
Maps of all tropical cyclone tracks in (a) V1 and (b) V2 of the model, and in the IBTrACS observations (c), from 1980 through 2000. Tracks are colored according to the storm's genesis region: North Indian (red), Western North Pacific (green), Eastern North Pacific (blue), North Atlantic (teal), South Indian (magenta), Australian Region (coral), South Pacific (maroon), and South Atlantic (hot pink).

For much of this analysis, in addition to global statistics, we also show statistics of TCs grouped by hemisphere, and by 8 standard TC basins indicated by the track colors in Figure 1 and defined in Table S1 of Supporting Information S1. We omit plots of the South Atlantic for most statistics given the paucity of storms there. For most statistics, the storm is labelled according the storm genesis basin, with the exception of Accumulated Cyclone Energy (ACE), for which we label the storm basin according to its location at each snapshot. ACE is defined as the sum of the squared maximum sustained wind speeds over 34 knots in observations (Bell et al., 2000). For the model, we instead use Modified Accumulated Cyclone Energy (MACE), which does not require a wind speed over 34 knots to be counted, following Camargo et al. (2005) and Camargo et al. (2016).

## 3 Tropical Cyclone Climatology

Figure 1 shows maps of tropical cyclone tracks in the model from 1980 through 2000 in the two model versions and in the IBTrACS observations. Some features are readily apparent: both model versions under‐represent the observed number of TC tracks in most regions, especially in the North Atlantic and the eastern North Pacific, regions in which such low biases in TC activity are common across models and reanalysis (e.g., Aarons et al., 2021; Camargo, 2013; Camargo & Wing, 2016; Camargo et al., 2020; Roberts et al., 2020a). Conversely, both model versions have several TCs in the South Atlantic, while IBTrACS contains none in this region from 1980 to 2000. Historically, the South Atlantic has been considered a region free of TCs. However, since the occurrence of Hurricane Catarina (2004) and Subtropical Storm Anita (2010) (Dias Pinto et al., 2013; Pezza & Simmonds, 2005; Veiga et al., 2008), recent studies have shown the regular occurrence of TCs with subtropical characteristics there, similarly to the North Atlantic (Evans & Braun, 2012; Gozzo et al., 2014, 2017). There are generally more TCs in V2 than in V1, in all regions except the Eastern North Pacific.

**Figure 2:**
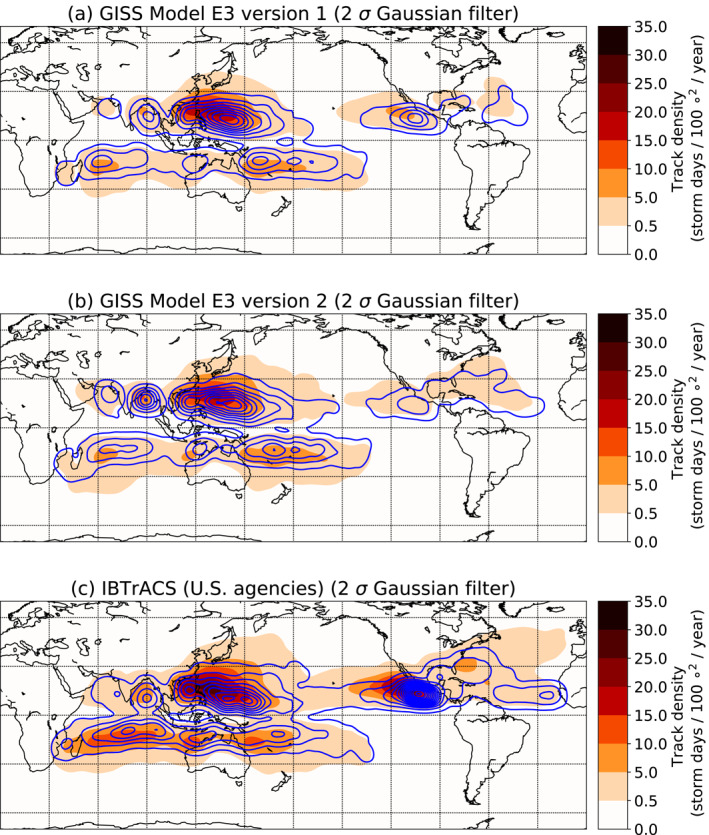
Maps of TC track density (colors) and genesis density (contours) in (a) V1 and (b) V2 of the model, and in the (c) IBTrACS observations, from 1980 through 2000. Contours are drawn at 0.1 and in increments of 0.5, starting from 0.5, in units of storm days per 100 square degrees per year. A Gaussian filter with a *σ* value of 2 has been applied to the densities to create smoother contours with small numbers of storms.

Figure 2 shows the density of storm tracks (colors) and genesis locations (contours) in each model version and in the observations, calculated as the number of storm‐days or genesis occurrences in the data set within the 10° by 10° box centered on any particular point, divided by the number of years. The biases seen in Figure 1 are also apparent here, as are the subtle changes from V1 to V2. The model versions capture many broad features of the spatial distribution of the observed storms, such as the local maxima in the track and genesis densities over the Western North Pacific, the Bay of Bengal, and off the coast of Mexico, but they mostly underestimate the magnitude of these features, especially in the Eastern North Pacific and North Atlantic.

**Figure 3:**
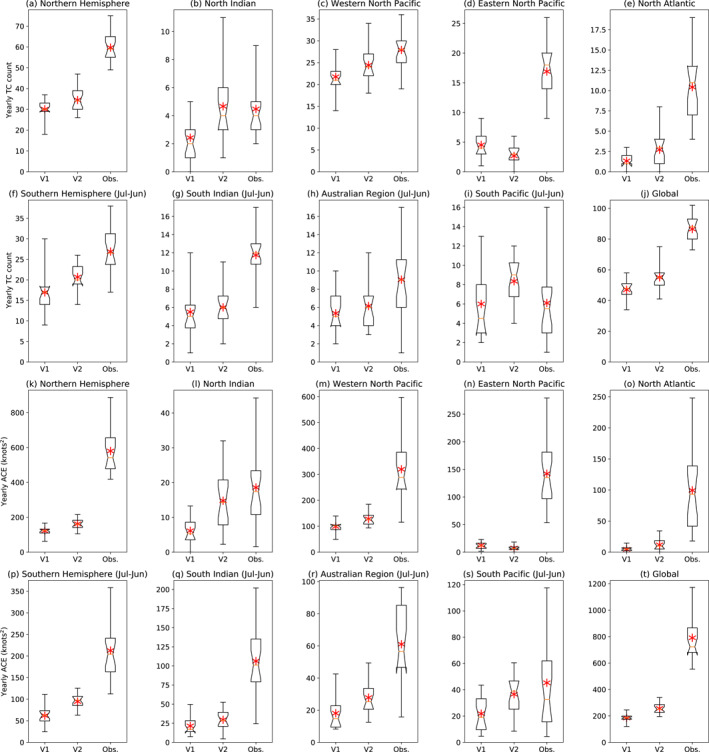
Box plots showing distributions of TC counts (top 10 panels) and ACE/MACE (bottom 10 panels) per year from 1980 through 2000 in V1, V2 and the observations, globally, by hemisphere, and by genesis region. Years defined from January to December for the Northern Hemisphere (21 years) and from July to June for the Southern Hemisphere (20 years, excluding the two half seasons). Red asterisk indicates the mean. Notch indicates 95% confidence interval of the median. Bounds of the boxes indicate first and third quartiles. Whiskers indicate the full range of data, with no provision for outliers.

Figures 3a–3j shows the distributions of the number of TCs per year globally, by hemisphere, and in each region, in each model version and the observations. Globally (Figure 3j), V2 has slightly more storms than V1, but both underestimate the observed number of TCs by roughly a factor of 2. The model underestimation is strongest in the Eastern North Pacific, North Atlantic, and South Indian regions, while V2 produces similar numbers to the observations in the North Indian Ocean and actually overestimates the observed TC counts in the South Pacific. The mean number of storms increases from V1 to V2 in every region except for the Eastern North Pacific, where V2 has even fewer TCs than V1.

Figures 3k–3t shows similar box plots but for ACE/MACE per season. The model generally underestimates this quantity relative to observations by more than it does the TC counts, indicating that the storms are weaker in the two model versions than in observations, as can be expected for models at this resolution (Davis, 2018; Moon, Kim, Camargo, Wing, Reed, et al., 2020; Roberts et al., 2020a) and which will be further explored in Section 4. Even without a minimum wind speed threshold to be counted towards MACE, the model underestimates ACE by about a factor of 4 globally (Figure 3t), and by an order of magnitude in the Eastern North Pacific and North Atlantic (Figures 3n and 3o), which arises mainly from the lack of strong TCs in the model. On the other hand, the high number of weak storms leads to similar values between V2 and the observations in the South Pacific (Figure 3s).

**Figure 4:**
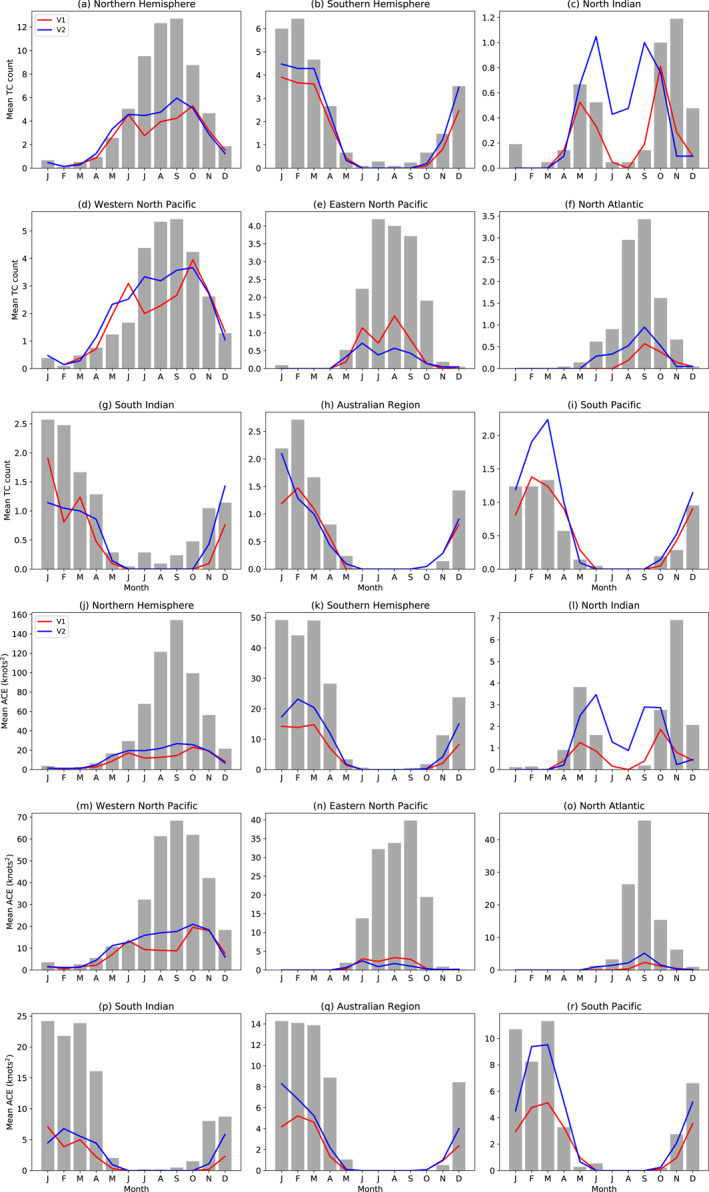
Mean number of tropical cyclones per year from 1980 to 2000 in each month, for the (a and b) two hemispheres and in (c–i) each of seven regions, in observations (bars) and the two model versions, and equivalent plots for ACE/MACE (j–r).

Figures 4a–4i shows the seasonal cycle of the number of tropical cyclones in each hemisphere (Figures 4a and 4b) and region (Figures 4c–4i). In both the Northern and Southern Hemisphere, the low bias in TC frequency is concentrated in the peak months of the TC season, with the model doing better during the early and late seasons and correctly simulating the lack of storms in the off season. Interestingly, in the North Indian Ocean, while V2 matches the observed total number of storms better than V1 does (Figure 3b), Figure 4c shows that these additional storms occur at the wrong time, during the summer monsoon. In reality, this basin has few tropical cyclones between the pre‐ and post‐monsoon peaks (e.g., Liu et al., 2021). This bias was previously documented in the GISS Model E2 (Camargo et al., 2016), as well as other models (Camargo, 2013; Camargo et al., 2005; Shaevitz et al., 2014), and two possible reasons were considered, first the tracking algorithm having trouble distinguishing between monsoon depressions and weak model TCs, second the model producing TCs in the wrong season in the North Indian Ocean. However, this bias is much smaller in either version of E3 than in E2 (cf., Figure 9 of Camargo et al. [2016]). V1 correctly reflects the lack of TCs in the North Indian ocean in midsummer, and more closely matches the spring and fall peaks in this basin, but both versions underestimate the spring and fall peaks (more so for V1 in the spring) when looking at ACE/MACE (Figure 4l). The excess storms in V2 in the South Pacific are confined to the months of February through April.

We also show in Figures 4j–4r the seasonal cycle of ACE (observations) and MACE (model) in each hemisphere and region. As with the annual box plots, the model biases in ACE are in most cases more pronounced than those in TC counts, due to compounding errors of storms being too few and too weak. The excess TC activity in V2 in the Indian summer monsoon is still apparent in the ACE plot (Figure 4l), while the high number of storms compensates for their low intensity in V2 in the South Pacific leading to similar ACE/MACE (Figure 4r).

## 4 Tropical Cyclone Physical Properties

### 4.1 Storm Intensity and Lifetime

**Figure 5:**
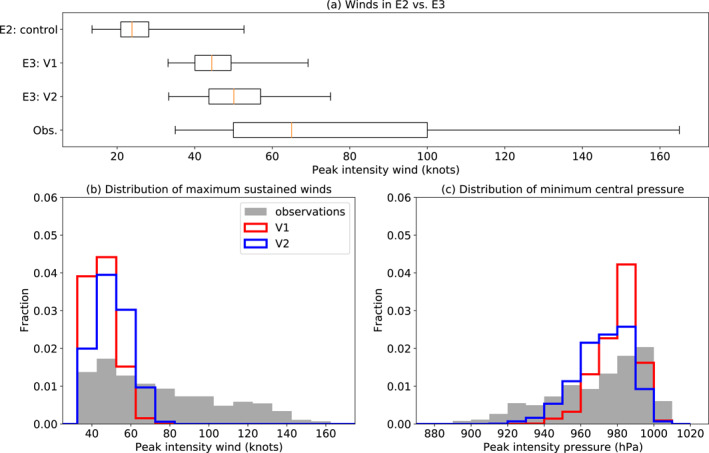
Distributions (a) of TC peak intensity sustained winds in the E2 control runs (Camargo et al., 2016), V1 and V2 of E3, and observations, and probability density functions of surface winds (b) and pressure (c) among TCs globally at peak intensity, in the observations (gray filled bars) and the two model versions (colored step plots). For the wind speed, each histogram bin includes two of the 5‐knot increments used in observational reporting.

Having explored the statistics of TC activity and seasonality, we now delve into the physical properties of the storms as simulated by E3, starting with the maximum surface (10 m) wind speed and minimum central pressure. Figure 5a shows distributions of the maximum wind speed of TCs in E2 at 1° resolution (results from Camargo et al. [2016]), versions V1 and V2 of E3, and observations. Recall that TCs in E3 must reach at least 17 m/s to be counted, whereas in E2 a wind speed threshold of 9 m/s was used in the Camargo and Zebiak (2002) tracking scheme to account for the inability of low‐resolution models to produce the wind speeds typical of real tropical cyclones (Davis, 2018; Moon, Kim, Camargo, Wing, Reed, et al., 2020; Walsh et al., 2007). E3 has sufficient resolution at 0.5° that a substantial number of tropical cyclones—albeit a substantially lower number per year than observed, as shown above—are found in the model even if we use the same threshold as in observations.

The maximum wind speeds have improved significantly from E2 to E3. The median TCs in E3 are of similar strength to those in the upper quartile for E2, and the strongest storms in E3 reach hurricane intensity (64 knots, 33 m/s). However, TCs in excess of 80 knots are still absent from E3. The further refinements of E3 from V1 to V2 led to still stronger TCs, but the improvement is much less than from E2 to E3. Notably, the median lifetime maximum wind speed of about 25 m/s in V2 is comparable to those of other GCMs at similar resolution (cf., Figure 8a of Shaevitz et al. [2014]), including the approximately 40 km High‐Resolution Hadley Center Global Environmental Model Version 3 (HadGEM3), 50 km HiRAM, and 56 km Goddard Earth Observing System Model Version 5 (GEOS‐5). This suggests that with E3, the GISS GCM is now competitive with other models in its TC representation, at least for tropical storms, with the caveats that the upper tail of wind speed distribution does not extend into Category 2 or 3 on the Saffir‐Simpson scale in E3 as it does in these other models. (In addition, the CMIP6 versions of these other models may contain subsequent improvements to their TC representations even at the same resolution, compared to what was shown in Shaevitz et al. [2014].)

Figures 5b and 5c show histograms of TC maximum wind speed and minimum central pressure at peak intensity, in V1, V2, and the observations, normalized by the number of storms in each data set. For the wind speed (Figure 5b), storms in both model versions are much weaker than those observed, with very few storms reaching hurricane intensity (exceeding 64 knots) in V1, and some Category 1 storms in V2 but still none exceeding 80 knots. The minimum central pressures, however (Figure 5c), show better model performance at simulating stronger storms, especially for V2, with some reaching as low as 920 hPa. Since the wind speed is related to the pressure gradient, the pressure gradient for a given minimum central pressure is related to storm size, and storm size is constrained by horizontal resolution, it is not surprising that, at this resolution, higher storm intensities are captured if pressure is used as a metric than if wind is. This characteristic has been noticed in many other models, for example, Roberts et al. (2015). Comparing the probability density functions in Figure 5b to those of the GCMs in Figure 7b of Roberts et al. (2020a) confirms that E3 simulates TCs achieving comparable intensity to those of similar resolution GCMs after a further 6 years of model development since Shaevitz et al. (2014). When using maximum wind speed as TC intensity, of the GCMs analyzed by Roberts et al. (2020a), only the 25 km resolution version of the Euro‐Mediterranean Centre on Climate Change coupled climate model (CMCC‐CM2‐VHR4; Cherchi et al., 2019) and the 50 km Centre National de Recherches Météorologiques version 6‐1 model (CNRM‐CM6‐1‐HR; Voldoire et al., 2019) have an appreciable fraction of storms of at least category 2 intensity, with the rest, like E3, having very few if any storms exceeding 80 knots.

**Figure 6:**
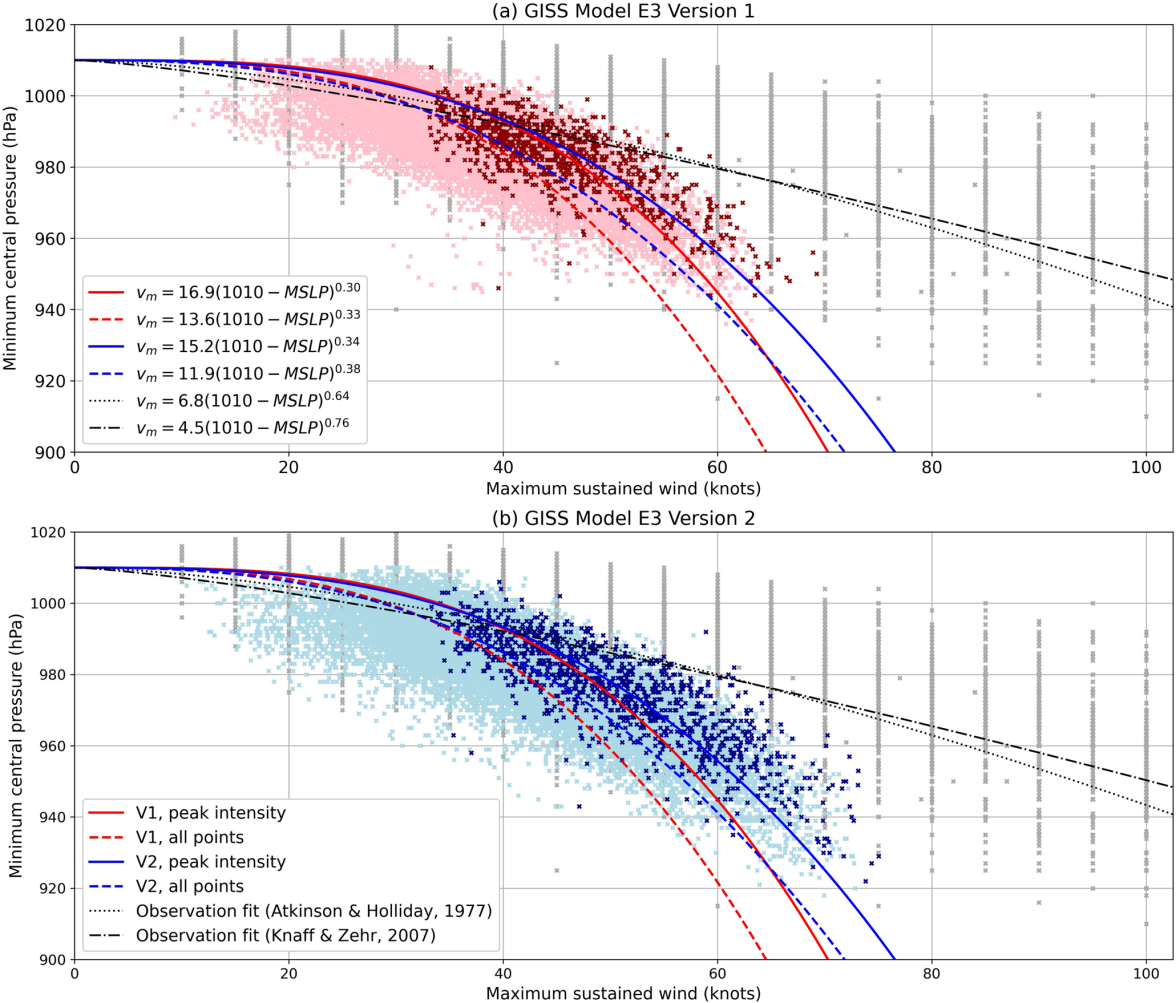
Scatter plots of maximum 10 m sustained wind versus minimum sea level pressure (MSLP) across all storms in (a) V1 and (b) V2, with lighter dots indicating all intensities during the storm's lifetime, and darker dots indicating the peak intensity as defined by maximum sustained wind. Power law fits to the modeled storms are shown as dashed and solid curves for the full set and peak intensity data points, respectively, in red for V1 and blue for V2. Wind and pressure observations from IBTrACS (all intensities, not peak intensity, up to 100 knots) are shown as gray dots, and empirically derived power law relations from observations (Atkinson & Holliday, 1977; Knaff & Zehr, 2007) are shown as black curves. A few model pressures greater than 1,010 hPa were omitted to avoid complex numbers in the fit calculation. Equations for the best fit curves are shown in the figure legend, where *v*_*m*_ is the storm's maximum sustained wind speed and MSLP is the mean sea level pressure.

Figure 6 shows the relationship between wind and pressure in each model version, including least squares power law fits to all data points and those at peak intensity, along with empirically derived relationships from observations (Atkinson & Holliday, 1977; Knaff & Zehr, 2007), similar to Figure 2 of Kim et al. (2018). The modeled storms generally do seem to follow a power law structure, especially at peak intensity, with the slope being shallower in V2 (cf., red and blue curves in Figure 6), indicating stronger winds for the same pressure. Both versions have a much steeper slope than the observations, consistent with the better agreement of the probability density functions for pressure than for wind speed shown in Figure 5.

**Figure 7:**
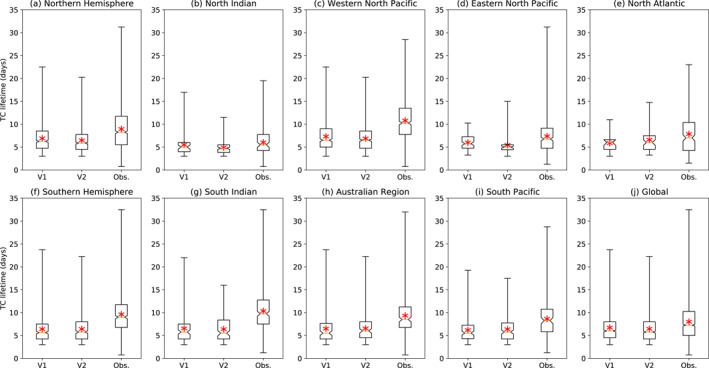
Box plots showing the distribution of TC lifetime in V1, V2, and observations, (j) globally, by (a and f) hemisphere, and by (b–e and g–i) genesis region. Mean value indicated by red asterisk; notch indicates 95% confidence interval for the median.

Figure 7 shows the distribution of TC lifetime in the model and observations, globally and in the different regions. The model only slightly underestimates the observed TC lifetimes, consistently across different regions, with no significant differences between V1 and V2, except in the Eastern North Pacific (Figure 7d) where TCs are about a day shorter‐lived in V2. The model also qualitatively reproduces some of the observed differences in average lifetime between regions, such as TCs being longer‐lived in the Western North Pacific (Figure 7c) than in the North Indian ocean (Figure 7b). Note that our storm tracker settings impose a minimum lifetime of 3 days for TCs to be counted, which is reflected in the box plots. We also made similar plots for the distributions of peak wind speed and pressure by region (not shown) and found that these quantities vary little by region.

### 4.2 Storm Structure

**Figure 8:**
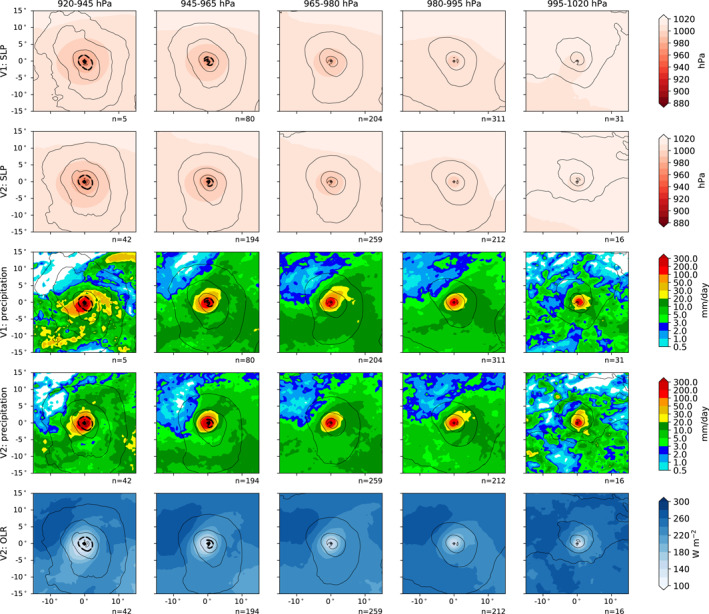
Composite, storm‐centered properties of Northern Hemisphere tropical cyclones, binned by peak intensity pressure. Contours show tangential velocity at 10 m, in 5 m/s intervals, with a thicker contour at 20 ms. Colors show: sea level pressure in V1 and V2 storms (top two rows); precipitation in V1 and V2 (third and fourth rows); and outgoing longwave radiation in V2 (fifth row). The number of storms in each pressure bin is indicated by *n*.

**Figure 9:**
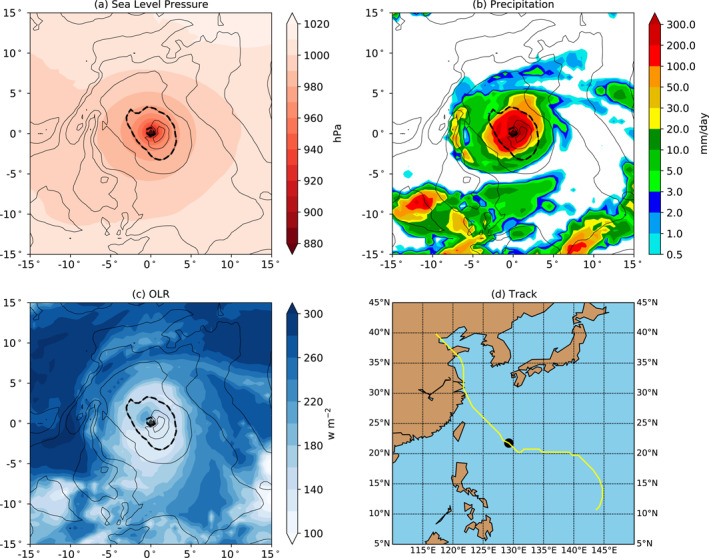
Tangential winds at 10 m (contours, interval 5 m/s) and (a) sea level pressure, (b) precipitation, and (c) OLR at peak intensity for the strongest storm by minimum central pressure in the V2 20‐year test, and (d) map of the track with the location at lowest pressure marked with a dot. Thicker dashed contour is at 20 m/s.

To better understand the physical properties of the storms, we show in Figure 8 several properties of TCs at peak intensity in a 15° by 15° box centered on the storm center, averaged over five pressure bins following Figure 8 of Roberts et al. (2020a). The <920 hPa bin is excluded because no storms reached that pressure in V1 and only one did in V2; we include this storm in Figure 9 as a case study. We include only storms in the Northern Hemisphere to better show any asymmetry between different quadrants. While the sea level pressure contours (colors, top two rows of Figure 8) are generally concentric and elliptical, the tangential winds (black contours, all rows) are more kidney‐shaped, indicating stronger winds north and east of the center, as would be expected given generally westward and northward motion at the times when storms are typically at peak intensity. Precipitation, meanwhile (third and fourth rows of Figure 8), is strongest in the southwest and northeast quadrants (at least in the core of heavy precipitation indicated by the 20 mm/day contour) and for the strongest storms, the precipitation maximum is west of the center. The precipitation minimum in the northwest quadrant is likely due to entrainment of subtropical and mid‐latitude dry air into the storm following its cyclonic and radially inward circulation. These spatial structures in precipitation are similar for V1 and V2, with the main difference between versions being greater numbers of storms in the higher intensity bins in V2. For V2, we also had the necessary (instantaneous, 6‐hourly) data available for outgoing longwave radiation (OLR), which is shown in the fifth row of Figure 8. This shows a similar pattern to precipitation of having the lowest OLR southwest and northeast of the center, which would be expected given the known correspondence between increased precipitation and decreased OLR.

The pattern of kidney‐shaped tangential wind contours and more concentric pressure contours is also seen in the modeled and reanalysis TCs shown in Roberts et al. (2020a), especially at the lower resolution they analyze. However, the equivalent wind speed contours are much closer to the center in our plots, indicating some combination of smaller storms and weaker winds at the same pressures. Vannière et al. (2020) and Zhang et al. (2021) also analyzed storm‐centered precipitation composites in multiple GCMs and in satellite‐derived rainfall rates (see their Figures 1 and 8–11, respectively), and found that a southwest‐to‐northeast orientation of the core of heavy precipitation is a common feature of Northern Hemisphere TCs in observations and in other GCMs. Zhang et al. (2021) showed that this orientation is flipped vertically for Southern Hemisphere TCs, and that, as in E3, there is more precipitation equatorward than poleward of the storm further away from the center, although interestingly this is flipped in observations for the strongest TCs in the Northern Hemisphere.

The strongest storm in the V2 20‐year integration, whose properties corresponding to Figure 8 are shown in Figures 9a–9c, provides a useful case study of a strong TC in E3 without averaging artifacts. This storm originates in the Western North Pacific region in June 1994 at 10.75°N, 143.75°E, as shown in the track map in Figure 9d, and reaches its lowest minimum central pressure of 918 hPa at 21.75°N, 129.25°E. The typhoon's peak wind speed of 73.8 knots is reached 6 hr after the time of its lowest pressure, at which point the maximum wind speed is 69.9 knots and the storm is moving west‐northwestward. At the time of its lifetime minimum pressure as shown in the figure, the storm exhibits a kidney‐shaped wind maximum east‐southeast of the center and a central minimum indicated by the reappearance of the 20 m/s contour. In this case precipitation (Figure 9b) is also maximized east of the storm center, but the broader core region of precipitation indicated by the 20 mm/day contour is aligned perpendicular to the axis of the 20 m/s wind contour, a similar pattern to that seen in Figure 8. Unlike the averages shown in Figure 8, this individual storm has a broad region of little to no precipitation (<0.5 mm/day) surrounding the core, while there are isolated areas of heavy precipitation to the south of the TC. This suggests that the broad swath of light precipitation surrounding the TCs in Figure 8 could be an artifact of averaging mesoscale precipitation features across different TCs. The OLR for this storm (Figure 9c) shows several interesting features: there is a local maximum of OLR, indicative of reduced cloud cover, which would be suggestive of an eye from an infrared satellite image, but it is centered northwest of the pressure minimum and wind speed maximum, suggesting that the vertical structure of the storm is tilted. There is also a band of cloud cover about 5° north of the storm that extends east to the edge of the plot, and corresponds with a streak of precipitation, suggesting that the model might be capturing some outer rainbands associated with TCs.

**Figure 10:**
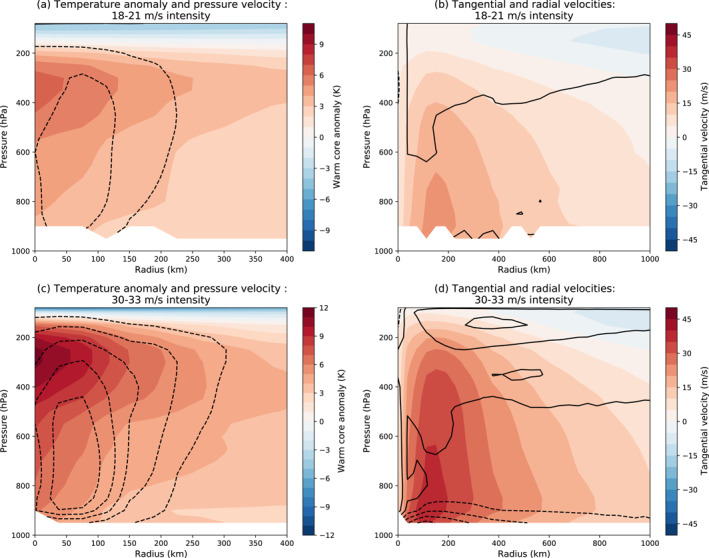
Azimuthally averaged temperature anomaly, pressure velocity, and tangential and radial velocity for TCs in V1 in 1994 and 1995, grouped into maximum sustained wind speed bins of (a and b) 18–21 m/s and (c and d) 30–33 m/s, following Kim et al. (2018) and Moon, Kim, Camargo, Wing, Sobel, et al. (2020). Contour interval is 0.3 Pa/s for pressure velocity and 3 m/s for radial velocity, with negative values dashed. Note different horizontal scales for the two columns.

We have also examined TC structure from a radial‐height perspective, following the methodology of Kim et al. (2018) and Moon, Kim, Camargo, Wing, Sobel, et al. (2020), who analyzed 8 different GCMs. Figure 10 shows azimuthally averaged temperature anomalies and pressure velocities (Figures 10a and 10c) and tangential and radial velocities (Figures 10b and 10d) for V1 TCs with maximum sustained winds between 18 and 21 m/s (Figures 10a and 10b) and between 30 and 33 m/s (Figures 10c and 10d), averaged across all 6‐hourly storm data points that fit into these wind speed bins in 1994 and 1995. These correspond to Figures 2 through 5 of Moon, Kim, Camargo, Wing, Sobel, et al. (2020). Temperature anomalies are defined as the mean within a 1,000‐km‐wide square centered on the storm, minus the mean inside a concentric 2,000‐km‐wide square but outside the smaller square, as in Kim et al. (2018). The warm core temperature anomaly structures are comparable to those in the models analyzed by Moon, Kim, Camargo, Wing, Sobel, et al. (2020), with maxima at the upper end of the range of those models. The updrafts, however, are weaker in V1 than in any of the Moon et al. models, for both wind speed bins. Consistent with this, radial profiles of precipitation (Figure S1 in Supporting Information S1) show that the precipitation maximum is weaker in GISS TCs than in other GCMs shown in Figure 6 of Moon, Kim, Camargo, Wing, Sobel, et al. (2020). Most GCMs, especially at higher resolutions, tend to produce more rain near the center of TCs than in satellite retrievals (Moon, Kim, Wing, et al., 2020) suggesting that the GISS‐E3 model may be closer to reality than the other models in terms of having weaker precipitation and updrafts. The vertical structures of the tangential velocities in V1 are similar to those of the other models for both intensity bins, as would be expected from thermal wind balance (or the analogous nonlinear balance appropriate for TCs) given similar thermal structures. Radial inflows near the surface are much weaker in V1 than in the Moon et al. models, never reaching the −3 m/s contour when the other models reach at least −6 and with the zero contour indeed hovering near the surface. For the 30–33 m/s storms, however, the surface inflow in V1 is similar in magnitude to the other GCMs. The upper level outflow is weaker in V1 than in the other GCMs for both intensity bins. The weaker updrafts and weaker radial winds compared to other models are qualitatively consistent given conservation of mass. The peak of the updrafts is located about 50 km away from the storm center in E3, in contrast to some GCMs in Moon, Kim, Camargo, Wing, Sobel, et al. (2020) in which the strongest updrafts are at the storm center, and more consistent with real TCs, where updrafts are strongest in the eyewall. We were not able to do these calculations for V2 because they require 6‐hourly 3D fields which were not saved for V2.

## 5 Sensitivity Tests

In this section, we present the results of various 1‐year experiments that were done with V1 and V2 of the model to aid in model development and explore the space of sensitivity of various tropical cyclone and other climate variables to a number of parameters. A total of 51 such experiments are examined for V1, and four for V2, in addition to the same year (1990) from the 20‐year runs with the default values of the parameters analyzed in the previous sections. The parameters changed in the experiments and their default values are listed in Table 1. These parameters include dimensionless multipliers for the entrainment rates for the more weakly and strongly entraining plumes, referred to as *ϵ*_1_ and *ϵ*_2_, respectively, in the two‐plume cumulus convection parameterization, originally described by DelGenio and Yao (1993), which was used in previous versions of the GISS GCM (Kelley et al., 2020; Schmidt et al., 2014) and has been retained in E3.

**Table 1:** Parameters Varied in Sensitivity Tests, Abbreviations, and Default Values

Parameter	Abbreviation	Default value
Entrainment rate multiplier for weaker‐entraining plume	*ϵ*_1_	0.2
Entrainment rate multiplier for stronger‐entraining plume	*ϵ*_2_	0.6
Divergence damping for sponge layer	Ds	0.03
Divergence damping for internal mode	Di	0.05
Divergence damping for external mode	De	0.02

**Figure 11:**
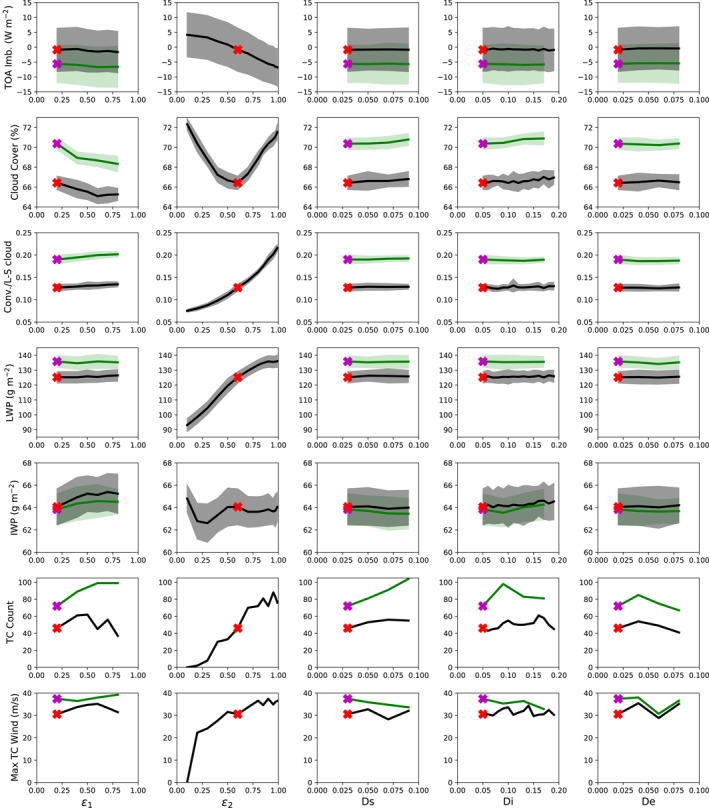
Results of 1‐year sensitivity tests conducted for V1 varying five different entrainment and damping parameters, including global means of, from top to bottom: TOA radiative imbalance, cloud fraction, ratio of convective to large‐scale cloud cover, liquid water path, ice water path, global TC count, and the strongest surface sustained wind for any TC. X symbol indicates default value, with the *y*‐value showing the global mean for year 1990 of the 20‐year test run. For non‐*ϵ*_2_ tests, black curves indicate tests using the default value of *ϵ*_2_, while green curves indicate tests repeated with *ϵ*_2_ = 0.9. Envelopes show the standard deviation across the 12 months.

Figure 11 illustrates the results of the V1 sensitivity tests of TC and climate variables to varying entrainment and damping parameters, in a similar format to Figure 3 of Mauritsen et al. (2012). The TC and climate variables shown include global TC counts (sixth row), the strongest observed TC winds (seventh row), and five global mean climate state variables: TOA radiative imbalance, cloud fraction, ratio of convective to large scale cloud fraction, liquid water path, and ice water path. Of the five model parameters shown, only *ϵ*_2_ has a systematic effect on TC properties, with higher values of *ϵ*_2_ associated with more and stronger TCs. Accordingly, many of the sensitivity tests were redone with *ϵ*_2_ increased to 0.9 from its default value of 0.6; these experiments are shown as green curves in Figure 11, whereas those with the default value of *ϵ*_2_ are shown in black. The experiments with *ϵ*_2_ = 0.9 consistently have higher TC counts and stronger TCs than those with *ϵ*_2_ = 0.6 across all of the values tried for the other parameters. However, increasing *ϵ*_2_ also affects other climate variables. Most importantly, the *ϵ*_2_ = 0.9 runs consistently have a TOA radiative imbalance of about −6 W m^−2^ (defined positive downward) in the annual mean, versus a default value that is close to 0, and this imbalance is insensitive to changing any of the other parameters. Increasing *ϵ*_2_ also leads to greater cloud cover (albeit decreasing *ϵ*_2_ from the default value also does this), a greater fraction of convective cloud, and a higher liquid water path, while having little effect on ice water path. Physically, these results are suggestive of a mechanism in which the increased entrainment rate leads to less deep convection, less convective subsidence drying, more shallow convection, a moister lower troposphere, and more clouds. The greater cloud cover causes a higher planetary albedo, which leads to the greater radiative imbalance, and the greater liquid water path makes TC formation easier.

Besides *ϵ*_2_, the other parameters have little systematic effect on any of the TC or climate variables. Most notably, we find little sensitivity to the divergence damping parameters. This is in contrast to Zhao et al. (2012), who found a strong sensitivity of TC properties to divergence damping parameters in the GFDL HiRAM model, and is somewhat surprising given that HiRAM uses the same dynamical core as E3 and was run at the same C180 resolution in Zhao et al. (2012). They found, as we do, that divergence damping parameters did not significantly affect the large‐scale state of the atmosphere, and they attribute the effect of these terms on TCs to suppression of convective noise at small spatial scales, which they suggest produces more favorable conditions for larger‐scale disturbances that generate TCs. We do not analyze convective variance spectra in this paper, but our results suggest that this mechanism is either absent in E3 or that other environmental factors are inhibiting additional large‐scale disturbances from actually developing into TCs. In the experiments where Ds was varied with *ϵ*_2_ set to 0.9 instead of 0.6 (green curves in Figure 11), higher values of Ds do result in progressively more TCs, which could be due to the mechanism put forward by Zhao et al. (2012) and suggests that it is contingent on other conditions favorable for TC formation being met.

**Figure 12:**
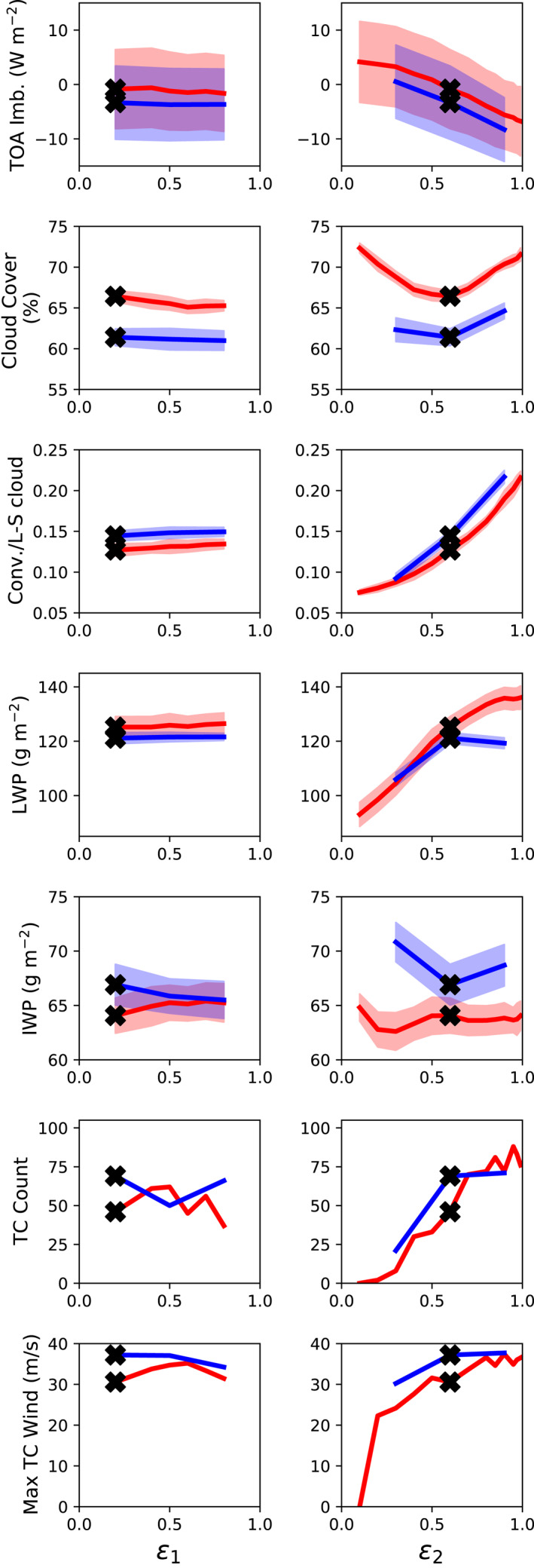
As in Figure 11 but for sensitivity tests conducted varying entrainment rate parameters in V2 (blue curves), compared with results varying the same parameters in V1 (red curves).

Experiments varying *ϵ*_1_ and *ϵ*_2_ were repeated for V2, and these results are plotted in Figure 12 (blue lines) along with the equivalent V1 experiments (red lines). In V2, decreasing *ϵ*_2_ to 0.3 reduces TC counts, but increasing it to 0.9 barely increases them. In fact, V2 with *ϵ*_2_ = 0.6 has as many TCs as V1 with *ϵ*_2_ = 0.9. At the default parameter values, V2 has a slightly larger TOA energy imbalance, lower cloud cover, greater fraction of convective cloud, lower liquid water path and higher ice water path relative to V1. These variables respond in similar ways to changes in *ϵ*_1_ and *ϵ*_2_ in V2 as in V1, except that in V2, raising *ϵ*_1_ reduces ice water path, and raising *ϵ*_2_ to 0.9 reduces liquid water path (a variable that generally seems to go with TC counts in these tests). Changes in liquid and ice water path tend to oppose each other in V2 to a greater degree than in V1.

**Figure 13:**
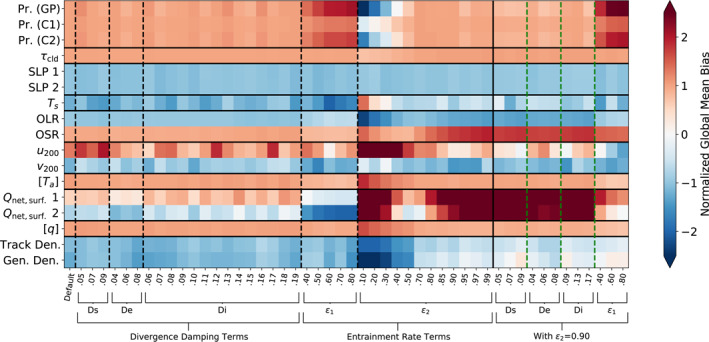
Errors in the V1 default 20‐year test (left column) and 1‐year sensitivity tests relative to observations, expressed as the model's overestimation (red) or underestimation (blue) of the global means of the observed quantities, normalized by dividing by the median of the absolute value for each row. Parameters listed in Table 1. Key to observations given in Table 2.

**Figure 14:**
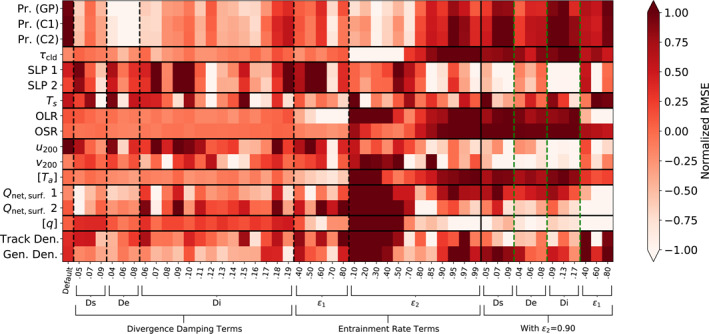
As in Figure 13 but for RMSE weighted by cosine of latitude, normalized by dividing by the inter‐quartile range of each row and subtracting the row median.

**Figure 15:**
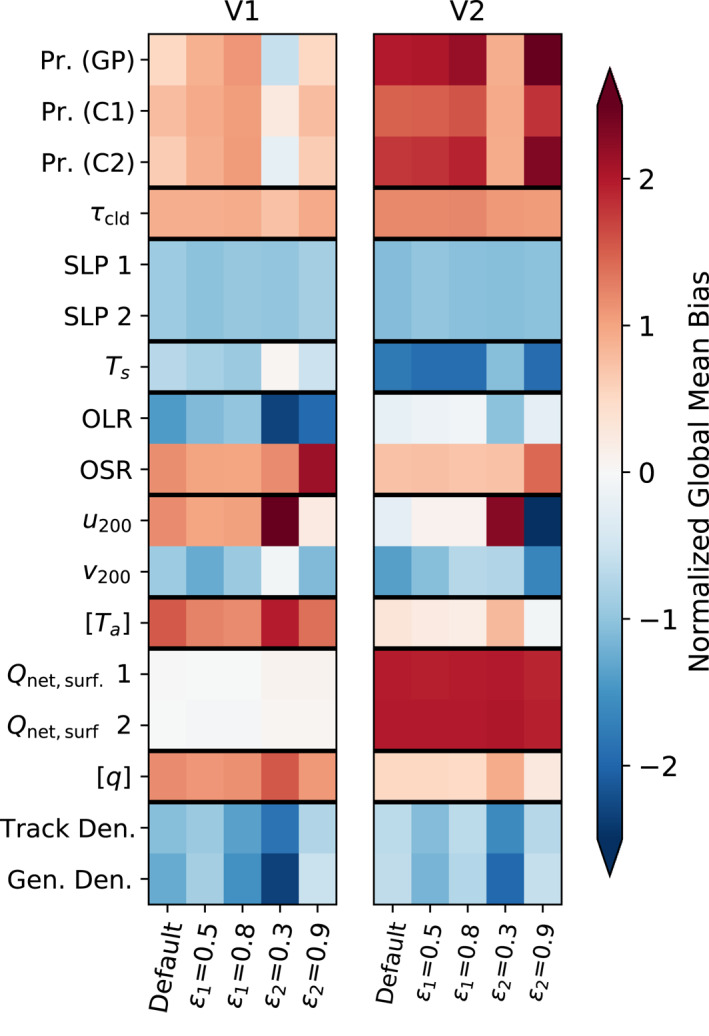
As in Figure 13 but for the sensitivity tests conducted for V2 and the equivalent tests also done for V1, with the normalization by median absolute value applied across the 10 experiments for each row.

**Figure 16:**
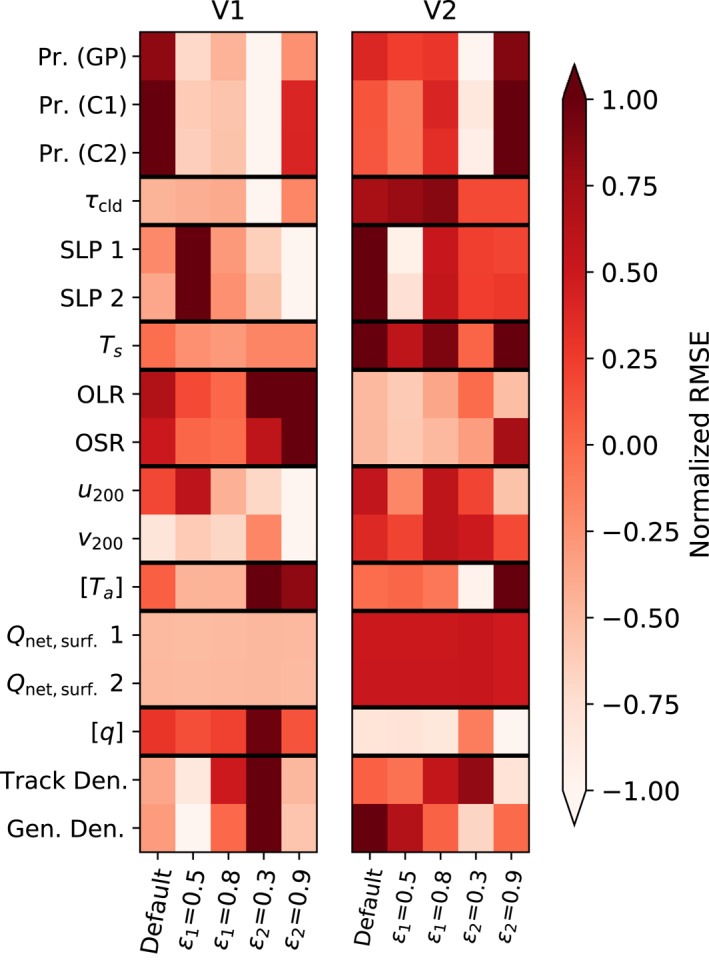
As in Figure 15 but for RMSE weighted by cosine of latitude, normalized by dividing by the inter‐quartile range of each row and subtracting the row median.

To further assess how climate and TC properties vary across these sensitivity tests in relation to each other and to observations, we plot matrices of error metrics in the V1 sensitivity tests relative to observations in Figures 13 and 14, and for V2 in Figures 15 and 16. Observation sources are given in Table 2. All observations are averaged across the years 1981 through 2010, except for the satellite‐derived quantities which were averaged over the shorter periods available: the International Satellite Cloud Climatology Project (ISCCP; July 1983 through December 2009) and Clouds and the Earth's Radiant Energy System (CERES; March 2000 through March 2018) data sets. Two different metrics of error are shown: the difference in the global mean of each quantity, or normalized bias (Figure 13), and the latitude‐weighted root‐mean‐square error (RMSE; Figure 14). Zonal mean height‐varying quantities are averaged across the pressure levels and latitudes with latitude area weighting. To better visualize the errors across different variables simultaneously, we apply a normalization for each variable: for the difference in global means, we divide by the median absolute value across all of the experiments for each variable, and for the RMSE, we divide by the inter‐quartile range (IQR) across the experiments and subtract the median. The latter normalization is similar to the method described by Gleckler et al. (2008) and used, for example, by Zhao et al. (2018) for visualizing relative error metrics across model experiments, except that we normalize RMSE by the IQR instead of the median in order to better draw out inter‐model differences when the median RMSE is large and the IQR is small. These normalizations allow us to easily see how changing a given tuning parameter affects the error for each variable, but they are not useful for comparing the magnitude of error across different variables. In our visualizations, for the normalized bias plots, blue colors indicate the model underestimates an observed quantity and red colors indicate the model overestimates it, with paler shades indicating better agreement. For the RMSE plots, darker colors indicate a greater RMSE.

**Table 2:** Observations Analyzed in RMSE Plots in Figures 14 and 15, With Data Sources Indicated

Label	Variable	Data source	Reference
Pr. (GP)	Precipitation	GPCP version 2.3	Adler et al. (2018)
Pr. (C1)	Precipitation	CMAP (standard)	Xie et al. (2007)
Pr. (C2)	Precipitation	CMAP (enhanced)	Xie et al. (2007)
*τ*_cld_	Cloud optical depth	ISCCP	Schiffer and Rossow (1983)
SLP 1	Sea level pressure over ocean	ERA‐I	Dee et al. (2011)
SLP 2	Sea level pressure over ocean	ICOADS	Freeman et al. (2017)
*T*_*s*_	Land surface air temperature	CRUTEM4 (standard)	Jones et al. (2012)
OLR	Outgoing LW radiation (TOA)	CERES EBAF Ed4.0	Loeb et al. (2018)
OSR	Outgoing SW radiation (TOA)	CERES EBAF Ed4.0	Loeb et al. (2018)
*u*_*200*_	200 hPa zonal wind	ERA‐I	Dee et al. (2011)
*v*_*200*_	200 hPa meridional wind	ERA‐I	Dee et al. (2011)
$\left[T_{a}\right]$	Zonal mean air temperature	ERA‐I	Dee et al. (2011)
*Q*_net, surf._ 1	Net surface heat flux	ISCCP, OAFlux	Schiffer and Rossow (1983) and Yu et al. (2008)
*Q*_net, surf._ 2	Net surface heat flux	CERES, OAFlux	Loeb et al. (2018) and Yu et al. (2008)
$\left[q\right]$	Zonal mean specific humidity	ERA‐I	Dee et al. (2011)
Track Den.	TC track density	IBTrACS	Knapp et al. (2010) and Kruk et al. (2010)
Gen. Den.	TC genesis density	IBTrACS	Knapp et al. (2010) and Kruk et al. (2010)

From Figure 13 we can see how the tuning parameters affect errors in global mean quantities while retaining information about the sign of the error. Tropical cyclone track density is consistently too low in the global mean, while increasing *ϵ*_2_ to 0.9 allows genesis density to sometimes exceed observations when other variables are changed (though the TCs are not necessarily in the correct locations). The model has a high bias in precipitation in most sensitivity tests, which is further increased by increasing *ϵ*_1_ and is only reduced by decreasing *ϵ*_2_ from the default value, which exacerbates the low bias in TCs frequency. (Note that some, e.g., Stephens et al. (2012), have argued that the satellite‐derived precipitation estimates to which we compare the model may have a low bias compared to reality, based on energy budget calculations.) The increase of precipitation with *ϵ*_1_ is consistent with the results of Kim et al. (2012), who changed the same parameter in E2 and found that it led to a high precipitation bias. Some variables, like cloud optical depth and sea level pressure, have consistent biases that are not much affected by changing the tuning parameters. The low *ϵ*_2_ tests reverse the sign of the land surface temperature bias in addition to that for precipitation, but also have larger biases in OLR, upper level zonal winds, zonal mean specific humidity, and surface radiation. In addition to the greater TOA radiative imbalance shown in Figure 11, the tests with higher *ϵ*_2_ have greater biases in the surface radiation imbalance, and the greater (negative) TOA imbalance is mainly due to too much outgoing shortwave radiation, consistent with the increase in cloud cover shown in Figure 11.

Measuring error by differences in global mean quantities does not account for the possibility that the model could get the global mean right but have the wrong spatial distribution. The normalized RMSE shown in Figure 14 accounts for this, albeit not considering error sign. Looking at the TC properties for the experiments where *ϵ*_2_ is increased from its default value, we see that while errors in the global means are small, in some cases RMSE is relatively large compared to other experiments. Given the small number of TCs in one year, errors in the spatial distribution especially for genesis density would be expected. However, the experiments with *ϵ*_2_ set to 0.9 generally have higher RMSE for precipitation and zonal mean atmospheric temperature than those with varying divergence damping terms and *ϵ*_2_ at its default value. Other variables, such as OLR and outgoing shortwave radiation (OSR), show more straightforward relationships between the two error metrics, with smaller global mean errors corresponding to smaller RMSEs, while some variables with little change in the global mean error across experiments, such as sea level pressure and cloud optical depth, have no apparent pattern in the normalized RMSEs due to amplification of noise by the RMSE normalization.

To see how these errors are affected by the change from V1 to V2, we show in Figure 15 the same difference in global means quantity as in Figure 13 but for the V2 default run and the four V2 sensitivity tests, along with their counterparts for V1. The median normalization is applied across this set of 10 experiments. Global TC track and genesis density errors are smaller in the V2 default run than in V1, with sensitivity tests producing little further improvement. Some climate variables, however, have greater global mean biases in V2 than in V1, particularly precipitation, land surface temperature, and the net surface heat flux. Interestingly, biases in both OLR and OSR are smaller in V2 than in V1, likely owing to the changes in the microphysics scheme in the case of OSR, but the TOA radiative imbalance shown in Figure 12 still increases since the compensation between these biases is reduced. The normalized RMSE metric (Figure 16) shows that errors in most climate variables (though not precipitation for the default case) are larger in V2 than in V1. RMSE values for TC track density and genesis density are also larger in the default case for V2 than V1, despite the number of tropical cyclones being higher. This is likely due to V2 having fewer TCs than V1 in the Eastern North Pacific (Figure 1), which is the place with the highest TC track and genesis density in the real world.

The similarity of V1 and V2 storms, combined with the relative insensitivity of TC variables to most tuning parameters and the common cube‐sphere dynamical core with E2, suggests that the improvement in TCs from E2 to E3 is primarily a result of increased resolution, but the improvements from V1 to V2, which retained the same default values of the entrainment and damping parameters, show that improving model physics can still lead to better representation of TCs. Future model runs with the final version of E3 at C180 resolution will likely show reduced biases in climate variables relative to V2, especially in the TOA radiation imbalance that was corrected in subsequent model development, with TC representation perhaps slightly further improved. It may also be possible to increase the number of TCs, as well as improve the representation of the seasonal cycle of TCs and reduce other climate biases, using stochastic parameterizations, as was done by Vidale et al. (2021) for two other GCMs. However, our results from V1 and V2 are already sufficient to show that E3 has substantially improved TC representation relative to E2, and that it performs in many ways comparably to other GCMs run at similar resolution.

## 6 Conclusions

In this study, we have explored the characteristics of the 0.5° resolution version of the GISS ModelE3 GCM, in two different versions across its development cycle. The representation of TCs is much improved from the CMIP5 GISS‐E2 GCM, with some TCs reaching hurricane intensity and the average storms in E3 having similar wind speeds to those of other half‐degree GCMs, obviating the need to use a tracker with a wind speed threshold well below that in observations. The model continues to show common biases in GCMs, such as having too few TCs, especially in the North Atlantic, and lacking storms of major hurricane intensity, although it is better at capturing more intense minimum central pressures than E2. The changes made between April 2018 (V1) and March 2019 (V2) further reduced biases in TC counts, intensities, and lifetimes, while also re‐introducing the excess of TCs in the North Indian basin during the summer monsoon (though to a much lesser degree than in E2). Our analysis of the simulated TCs' composite spatial structures shows that E3 reproduces spatial structures in winds, OLR and precipitation comparable to those seen in other models at similar resolution. Our analysis of azimuthally averaged TC properties (Figure 10) shows that the thermal structure of the simulated TCs is similar to that in other GCMs shown by Moon, Kim, Camargo, Wing, Sobel, et al. (2020), but updrafts, peak precipitation, and radial flows are weaker in E3 than in other GCMs for storms with equivalent intensity.

Our various sensitivity tests show that the most important parameter affecting TC numbers and intensity in E3 is the entrainment rate constant for the strongly entraining plume in the two‐plume convection scheme. Increasing this parameter, *ϵ*_2_, leads to more TCs. It also introduces greater errors in global mean energy imbalances at the surface and top of atmosphere, but experience suggests that these can be largely reduced by adjustments to other parameters, such as ice fall speed, without losing the benefits gained by increasing entrainment. We did not find divergence damping terms to be important for TCs, in contrast with experiments with the GFDL model by Zhao et al. (2012), who found that increasing the divergence damping parameter consistently increased TC counts, while increasing cumulus mixing rates caused TC counts to first increase then decrease. Kim et al. (2012) found that changing the convection scheme in E2 to a one‐plume scheme with an entrainment rate multiplier of 0.6 (in contrast to the control two‐plume experiment with *ϵ*_1_ = 0.3 and *ϵ*_2_ = 0.6) led to reduced TC counts, but subsequently increasing rain reevaporation dramatically increased them. Our results suggest that retaining the two‐plume scheme and changing the evaporation rate of the more strongly entraining plume instead of the weaker one provides a more useful, singular “knob” to optimize TC properties, without the need to change rain evaporation rates. Overall, biases in most climate variables across the sensitivity tests are slightly larger in V2 than in V1, but with both versions being snapshots in a years‐long process of model development that has not neared its end—particularly as there is no C180 version of Model E3 in the CMIP6 ensemble—it would be wrong to draw any inference of a trajectory in these errors from just these two versions.

Overall, we can conclude that the E3 version of the GISS GCM, when run at approximately 0.5° resolution, has much improved representation of TCs from the previous generation, and is now comparable to other GCMs of similar resolution, at least according to the average intensity storms. This indicates that E3 will be useful for future studies of TC responses to climate variability and change, whether on its own or as part of multi‐model ensembles.

## Supporting information

Supporting Information S1

## Data Availability

The IBTrACS observed TC data are available at https://www.ncdc.noaa.gov/ibtracs/index.php?name=ib-v4-access. Observational data from the GPCP, CMAP, ICOADS, and CRUTEM4 data sets listed in Table 2 are available at https://psl.noaa.gov/data/gridded/index.html. ISCCP data are available at https://isccp.giss.nasa.gov. ERA‐Interim reanalysis data are available at https://www.ecmwf.int/en/forecasts/datasets/reanalysis-datasets/era-interim. CERES data are available at https://ceres.larc.nasa.gov/data/. OAFLUX data are available at https://oaflux.whoi.edu/data-access/. Jupyter (Russotto et al., 2021) notebooks used to do the analysis and data files necessary to reproduce figures are posted online at https://doi.org/10.5281/zenodo.5542663.
