# Supplementary material for: Evolution of Tropical Cyclone Properties Across the Development Cycle of the GISS‐E3 Global Climate Model
Source: J Adv Model Earth Syst. 2022 Jan 5;14(1):e2021MS002601. doi: 10.1029/2021MS002601 (PMC9286432; doi:10.1029/2021MS002601)
Supplement: Supplementary file 1 — Supporting Information S1 [file JAME-14-0-s001.pdf]

JOURNAL OF GEOPHYSICAL RESEARCH

Supporting Information for "Improved Representation of Tropical Cyclones in the NASA GISS-E3 GCM"

Rick D. Russotto[1] *, Jeffrey D.O. Strong[1] †, Suzana J. Camargo[1], Adam Sobel[1,2], Gregory S. Elsaesser[2,3], Maxwell Kelley[3,4], Anthony Del Genio[3], Yumin Moon[5], and Daehyun Kim[5]

[1]Lamont-Doherty Earth Observatory, Columbia University, Palisades, NY, USA

[2]Department of Applied Physics and Applied Mathematics, Columbia University, New York, NY, USA

[3]NASA Goddard Institute for Space Studies, New York, NY, USA

[4]SciSpace LLC, New York, NY, USA

[5]Department of Atmospheric Sciences, University of Washington, Seattle, WA, USA

Contents of this file

1. Table S1
2. Figure S1

---

*Current address, Gro Intelligence, New York, NY, USA

†Current address, AIR Worldwide, Boston, MA, USA

References

Moon, Y., Kim, D., Camargo, S. J., Wing, A. A., Sobel, A. H., Murakami, H., . . . Zhao, M. (2020a). Azimuthally Averaged Wind and Thermodynamic Structures of Tropical Cyclones in Global Climate Models and Their Sensitivity to Horizontal Resolution. *Journal of Climate*, *33*(4), 1575-1595. doi: 10.1175/JCLI-D-19-0172.1

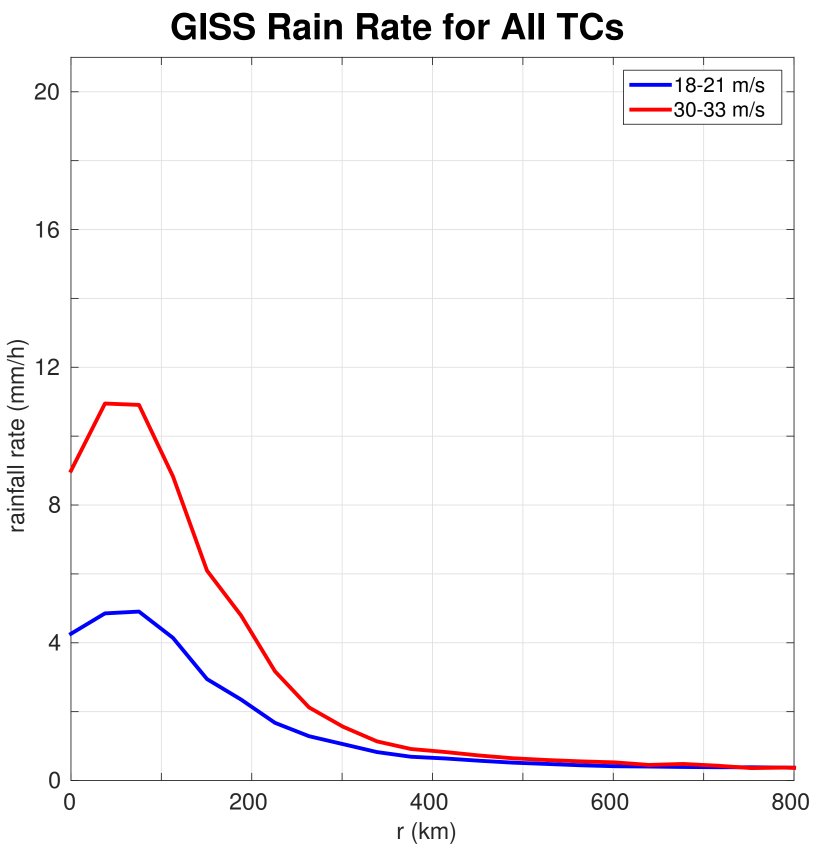

Figure S1. Radial profiles of rain rate for TCs in V1, binned by maximum wind speed. Compare to Figure 6 of Moon et al. (2020a).

Table S1. Definitions of regions used for tropical cyclone statistics (ϕ is latitude)

Region	Hemisphere	Western Boundary	Eastern Boundary
North Indian	Northern	35°E	99°E ($\phi > 8$°N)
		35°E	(105-$\frac{3}{4}\phi$)°E ($\phi \leq 8$°N)
Western North Pacific	Northern	99°E ($\phi > 8$°N)	160°W
		(105-$\frac{3}{4}\phi$)°E ($\phi \leq 8$°N)	160°W
Eastern North Pacific	Northern	160° W	107°W ($\phi > 24$°N)
		160° W	(65+$\frac{7}{4}\phi$)°W ($\phi \leq 24$°N)
North Atlantic	Northern	107°W ($\phi > 24$°N)	0°
		(65+$\frac{7}{4}\phi$)°W ($\phi \leq 24$°N)	0°
South Indian	Southern	25°E	105°E
Australian Region	Southern	105°E	165°E
South Pacific	Southern	165°E	70°W
South Atlantic	Southern	70°W	0°
